# Molecular characterization of *Streptococcus pneumoniae* causing disease among children in Nigeria during the introduction of PCV10 (GSK)

**DOI:** 10.1099/mgen.0.001094

**Published:** 2023-09-15

**Authors:** Stephanie W. Lo, Paulina A. Hawkins, Binta Jibir, Fatimah Hassan-Hanga, Mahmoud Gambo, Rasaq Olaosebikan, Grace Olanipekun, Huda Munir, Nicholas Kocmich, Amy Rezac-Elgohary, Safiya Gambo, Danstan Bagenda, Paul Fey, Robert F. Breiman, Lesley McGee, Stephen D. Bentley, Stephen K. Obaro, Community Acquired Pneumonia and Invasive Bacterial Disease (CAPIBD) Consortium, Zainab Adamu Haruna, Sadiq Abdussalam Ibrahim, Salisu Ya'u Sulaiman, Shamsuddeen Sani, Munir Sani, Sadiya Muhammad Sagagi, Sani Abdullahi Yunusa, Ubaidullah Nour Hanga, Aliyu Baban Maryam, Rukayya Nasir Sani, Jamila Bashir, Abubakar Shehu Gezawa, Muhammad Abdullahi Umar, Habiba Isah Ladu, Musa Abdullahi Zango, Fatima Ele Aliyu, Zakiyya Sadiq Abubakar, Abdurrauf Sani Yahaya, Muhammad Bello, Bilkisu Badamasi, Hajara Kabir Shehu, Kabiru Hamisu Haruna

**Affiliations:** ^1^​ Parasites and Microbes, Wellcome Sanger Institute, Hinxton, UK; ^2^​ Centers for Disease Control and Prevention, Atlanta, GA, USA; ^3^​ Hasiya Bayero Pediatric Hospital, Kano, Nigeria; ^4^​ Aminu Kano Teaching Hospital, Kano, Nigeria; ^5^​ Department of Pharmacology and Experimental Therapeutics, Thomas Jefferson University, Philadelphia, PA, USA; ^6^​ International Foundation against Infectious Diseases in Nigeria, Abuja, Nigeria; ^7^​ Division of Pediatric Infectious Disease, University of Nebraska Medical Center, Omaha, NE, USA; ^8^​ Murtala Muhammad Specialist Hospital, Kano, Nigeria; ^9^​ Department of Anesthesiology, University of Nebraska Medical Center, Omaha, NE, USA; ^10^​ University of Nebraska Medical Center, Department of Pathology and Microbiology, Omaha, Nebraska, USA; ^11^​ Emory Global Health Institute, Emory University, Atlanta, GA, USA; ^12^​ Rollins School Public Health, Emory University, Atlanta, GA, USA; ^13^​ Pediatric - Infectious Disease, School of Medicine, The University of Alabama, Birmingham, AL, USA

**Keywords:** genomics, Nigeria, pneumococcal conjugate vaccine, *Pneumococci*

## Abstract

*
Streptococcus pneumoniae* (*pneumococcus*) is a leading vaccine-preventable cause of childhood invasive disease. Nigeria has the second highest pneumococcal disease burden globally, with an estimated ~49 000 child deaths caused by pneumococcal infections each year. Ten-valent pneumococcal conjugate vaccine (GSK; PCV10) was introduced in December 2014 in a phased approach. However, few studies have characterized the disease-causing pneumococci from Nigeria. This study assessed the prevalence of serotypes, antibiotic susceptibility and genomic lineages using whole genome sequencing and identified lineages that could potentially escape PCV10 (GSK). We also investigated the potential differences in pneumococcal lineage features between children with and without sickle cell disease. A collection of 192 disease-causing pneumococcal isolates was obtained from Kano (*n*=189) and Abuja (*n*=3) states, Nigeria, between 1 January 2014 and 31 May 2018. The majority (99 %, 190/192) of specimens were recovered from children aged 5 years or under. Among them, 37 children had confirmed or traits of sickle cell disease. Our findings identified 25 serotypes expressed by 43 Global Pneumococcal Sequence Clusters (GPSCs) and 85 sequence types (STs). The most common serotypes were 14 (18 %, *n*=35), 6B (16 %, *n*=31), 1 (9 %, *n*=17), 5 (9 %, *n*=17) and 6A (9 %, *n*=17); all except serotype 6A are included in PCV10 (GSK). PCV10 (SII; PNEUMOSIL) and PCV13 formulations include serotypes 6A and 19A which would increase the overall coverage from 67 % by PCV10 (GSK) to 78 and 82 %, respectively. The pneumococcal lineages were a mix of globally spreading and unique local lineages. Following the use of PCV10 (GSK), GPSC5 expressing serotype 6A, GPSC10 (19A), GPSC26 (12F and 46) and GPSC627 (9L) are non-vaccine type lineages that could persist and potentially expand under vaccine-selective pressure. Approximately half (52 %, 99/192) of the pneumococcal isolates were resistant to the first-line antibiotic penicillin and 44 % (85/192) were multidrug-resistant. Erythromycin resistance was very low (2 %, 3/192). There was no significant difference in clinical manifestation, serotype prevalence or antibiotic resistance between children with and without traits of or confirmed sickle cell disease. In summary, our findings show that a high percentage of the pneumococcal disease were caused by the serotypes that are covered by currently available vaccines. Given the low prevalence of resistance, macrolide antibiotics, such as erythromycin, should be considered as an option to treat pneumococcal disease in Nigeria. However, appropriate use of macrolide antibiotics should be vigilantly monitored to prevent the potential increase in macrolide resistance.

## Data Summary

Genome sequences are deposited in the European Nucleotide Archive (ENA); accession numbers and a phylogenetic snapshot are available at https://microreact.org/project/GPS_Nigeria. Metadata of the pneumococcal isolates in this study have been submitted as a supplementary file. The authors confirm all supporting data, code and protocols have been provided within the article or through supplementary data files.

**Impact Statement**

Despite the high pneumococcal disease burden in Nigeria, isolating *Streptococcus pneumoniae* from clinical specimens has been a great challenge due to prior use of antibiotics and lack of microbiological expertise and/or reagents in laboratories. This study not only reported a relatively large number of disease-causing *S. pneumoniae* circulating in Nigeria, but also provides a comprehensive molecular characterization through whole genome sequencing. This study highlights the high percentage of disease-causing isolates covered by currently available vaccines and an effective treatment option, macrolide antibiotics, such as erythromycin, for pneumococcal disease. The identification of similar content of serotype, lineages and antimicrobial resistance between children with and without traits of or confirmed sickle cell disease revealed that the same preventive measure and treatment could potentially be used in both groups. Continued monitoring of the lineages expressing non-PCV10 (GSK) serotypes and with multidrug resistance such as GPSC5, 10, 26 and 627 is needed post-PCV introduction.

## Introduction

*Streptococcus pneumoniae* (pneumococcus) is a leading vaccine-preventable cause of childhood disease including pneumonia, sepsis and meningitis. It was estimated that nearly 9 million pneumococcal disease cases occurred in children aged under 5 years worldwide in 2015 and were responsible for 317 000 deaths [1 ]. Globally, Nigeria is one of the top four countries with the highest disease burden and mortality rate. It is estimated that pneumococcal disease causes 49 000 child deaths annually [1 ]. Together with India, Pakistan and the Democratic Republic of the Congo, these four countries accounted for approximately 50 % of global pneumococcal deaths [1 ].

Pneumococcal conjugate vaccines (PCVs) have proved to effectively reduce invasive pneumococcal disease (IPD) in children in many countries [2–7 ]. In Nigeria, a phased introduction of 10-valent PCV (PCV10; GSK), targeting ten pneumococcal serotypes that accounted for most of the disease in children, was launched in December 2014 with the support from Gavi, The Vaccine Alliance. PCV10 (GSK) was administered using a 3+0 vaccine schedule and the national PCV uptake rate was 36 % in 2015 and up to 52 % in 2019 according to a World Health Organisation monitoring system [8 ]. A study conducted between 2010 and 2016 serotyped 28 pneumococcal samples recovered from cerebrospinal fluid (CSF) using real-time PCR [9], and revealed that almost half of them (13/28) expressed serotypes targeted by PCV10 (GSK), while a number of them (*n*=14) were non-typeable by PCR [9 ]. A whole-genome sequencing study may provide further insight into the serotype distribution and their corresponding pneumococcal lineages.

Sickle cell disease is one of the major underlying conditions that increases the risk of developing IPD in children [10 ]. Nigeria has the highest birth prevalence of sickle cell disease in the world, with an estimated 150 000 births of babies with sickle cell anaemia annually [11 ]. Infants and children with sickle cell disease often have dysfunctional antibody production and poor opsonophagocytosis, making them more susceptible to pneumococcal disease [12 ]. As a preventive measure, penicillin prophylaxis and pneumococcal vaccines are recommended [13, 14 ].

In this study, we undertook whole-genome sequencing (WGS) on a collection of 192 disease-causing pneumococcal isolates collected from young children in Abuja and Kano, Nigeria, 2014–2018, as part of the Global Pneumococcal Sequencing (GPS) project [15]. We aimed to characterize the lineages, serotypes and antibiotic resistance of *S. pneumoniae* circulating in Nigeria. The data generated provides evidence to support future pneumococcal disease prevention and treatment strategies.

## Methods

### Bacterial isolates

This study enrolled children aged less than 5 years who presented to five government hospitals in Kano State (*n*=3), Nasarawa State (*n*=1) and Federal Capital Territory (*n*=1). In Kano state, the enrolment sites included the only tertiary healthcare facility, the largest Government hospital and a children’s hospital. Children with clinical signs that were suggestive of sepsis, pneumonia or meningitis between 1 January 2014 and 31 May 2018 were included. Specimens collected from enrolled children were subject to blood and/or CSF culture and the collected blood specimens were also used for haemoglobinopathy screening. During the study period, two children aged 6 and 10 years who were critically ill and their samples were subject to blood and CSF culture. The pneumococcal isolates collected from these two older children were also included in this study. These specimens were processed for culture at the International Foundation Against Infectious Diseases in Nigeria (IFAIN) Laboratory at Aminu Kano Teaching Hospital. *S. pneumoniae* was identified using standard microbiological methods. Haemoglobinopathy screening was performed using HPLC at the IFAIN central laboratory in Abuja. Sickle cell disease was defined as individuals with homozygous HbS (Hb SS) or haemoglobin variants other than HbA. Patients with sickle cell traits are individuals who had heterozygous HbS (Hb AS).

Ethical approval was obtained for enrolment in the clinical surveillance which required blood and CSF sampling, as clinically indicated, for bacteriological culture, further analysis of clinical specimens and management. The IRB references are Federal Capital Territory Health Research Ethics Committee: FHREC/2013/01/27/25-07-13; Aminu Kano Teaching Hospital: NHREC/21/08/2008/AKTH/EC/872 and AKTH/MAC/SUB/12 A/P-3/VI/972; FCT Health and Human Services Secretariat, FCTA/HHSS/NH/GEN/54/II/128; and University of Nebraska Medical Center, Omaha, NE, USA: 444-12-EP.

Overall, we collected disease-causing isolates from Kano (*n*=189) and Abuja (*n*=3) (retrieved from the Community Acquired Bacteremic Syndromes in Young Nigerian Children project [16 ]) between 2014 and 2018 (Fig. S2, available in the online version of this article).

### Genome sequencing and analysis

The pneumococcal isolates were sequenced on an Illumina HiSeq platform to produce paired-end reads of 151 bp in length and raw data were deposited at the European Nucleotide Archive (ENA) (Supplementary metadata). WGS data were processed as previously described [17 ]. Briefly, we inferred serotype and resistance for 16 antibiotics, including penicillin (PEN), amoxicillin (AMO), meropenem (MER), cefotaxime (TAX), ceftriaxone (CFT), cefuroxime (CFX), erythromycin (ERY), clindamycin (CLI), quinupristin-dalfopristin (SYN), linezolid (LZO), cotrimoxazole (COT), tetracycline (TET), levofloxacin (LFX), chloramphenicol (CHL), rifampin (RIF) and vancomycin (VAN) from the genomic data using SeroBA (version 1.0.0) [18 ] and a resistance detection pipeline developed by CDC [19–23 ]. The predicted minimum inhibitory concentration (MIC) values were interpreted according to Clinical Laboratory Standards Institute (CLSI, 2014, M100-S24) breakpoints. Penicillin resistance was defined as MIC of ≥0.12 µg ml^−1^, according to meningitis break-points. Our previous study showed high concordance between phenotypic and genotypic results of serotypes and antibiotic resistance [17 ]. Among the antibiotics, six commonly used antibiotics, penicillin, chloramphenicol, cotrimoxazole, erythromycin and tetracycline, were used for analysis of resistance patterns.

A pneumococcal lineage was defined by assigning a Global Pneumococcal Sequence Cluster (GPSC) to each isolate using PopPUNK (version 2.6.0) [24 ] and a reference list of pneumococcal isolates (*n*=42163, version 6) [25 ]. In addition, a multilocus sequence type (MLST) was derived from the genome data using MLSTcheck (version 2.1.1706216) [26 ]. Phylogenetic analysis was performed on all isolates by reconstructing a maximum likelihood tree using FastTree (version 2.1.10) [27 ] based on SNPs extracted from an alignment generated by mapping reads to the reference genome of *S. pneumoniae* ATCC 700669 (NCBI accession number FM211187) using Smalt (version 0.7.4). The metadata (supplementary file 1) and analysis results can be interactively visualized online using the Microreact tool at https://microreact.org/project/GPS_Nigeria.

### Statistical analysis

Categorical variables such as clinical manifestation, underlying condition (e.g. sickle cell disease), serotypes and GPSCs were compared using Fisher’s Exact Test while continuous variables such as age were compared by Student’s t-test. Before carrying out any Fisher’s Exact test, we calculated the number of samples that we needed to achieve an 80 % statistical power with a significant level of *P*<0.05 using the R package pwr which contains functions for basic power calculation [28 ]. When the variables do not have the sufficient statistical power to be tested, Fisher’s Exact Test was not performed. Multiple testing was adjusted using the Benjamin–Hochberg false discovery rate of 5 %. Statistical analysis was carried out in R version 3.5.2.

## Results and discussion

### Bacterial collection

The majority (99 %, 190/192) of samples were recovered from children aged 5 years or under, one from a child aged 6 years and the other from a child aged 10 years. Among them, 28 individuals had confirmed sickle cell disease and nine had the trait of sickle cell disease. The clinical manifestations were pneumonia (*n*=67), bacteraemia (*n*=53), meningitis (*n*=40), sepsis (*n*=28), upper respiratory tract infection (*n*=1) and unknown (*n*=3).

### Serotypes and vaccine coverage

**Fig. 1.**
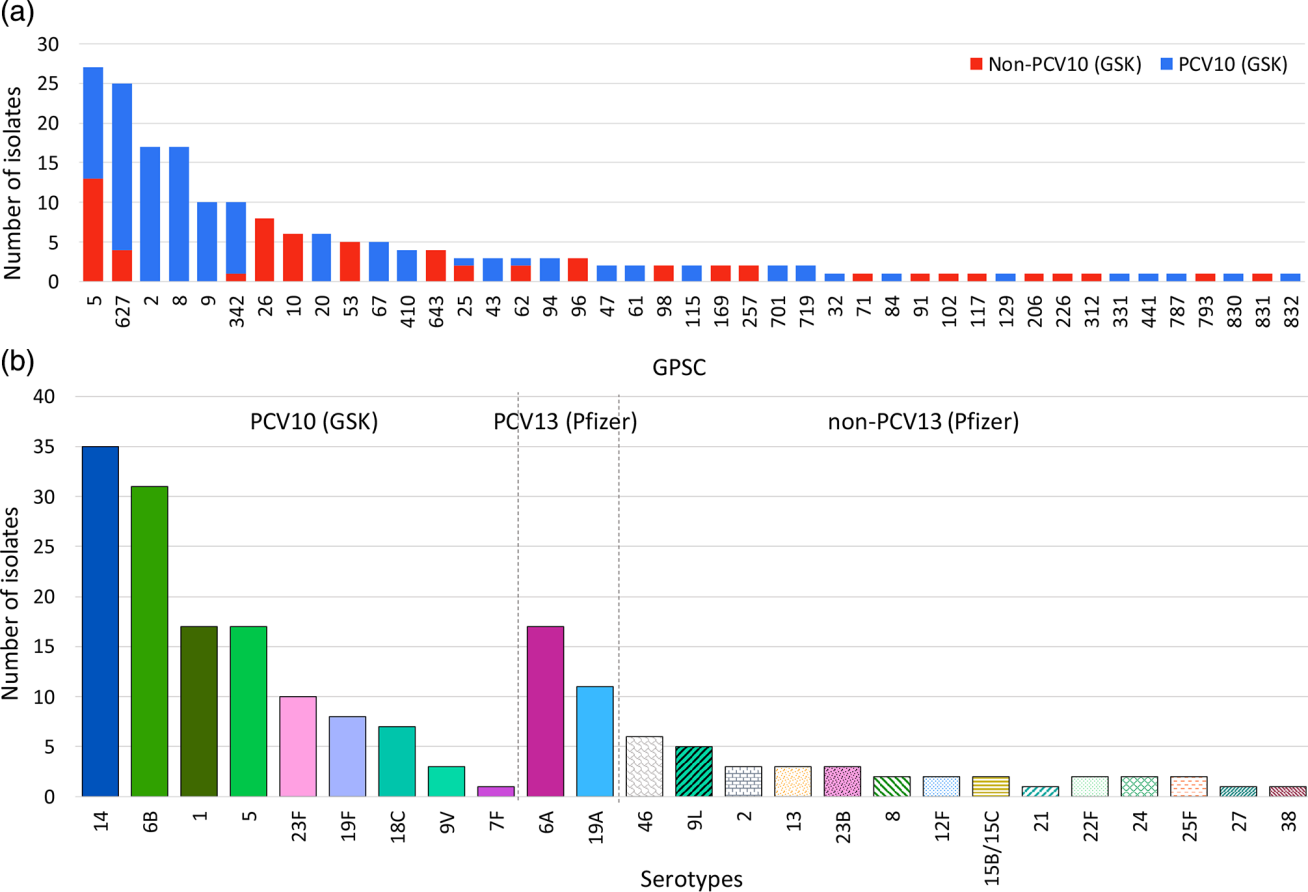
The distribution of GPSCs by PCV10 (GSK) and non-PCV10 serotypes (**a**) and the prevalence of serotypes (**b**) in a collection of 192 pneumococcal isolates from Nigeria, 2014–2018.

Serotypes predicted from WGS data revealed 25 serotypes expressed by 43 pneumococcal lineages (or GPSCs) that encompassed 85 sequence types (STs) (Fig. 1). The five most prevalent serotypes were serotype 14 (18 %, *n*=35), 6B (16 %, *n*=31), 1 (9 %, *n*=17), 5 (9 %, *n*=17) and 6A (9%, *n*=17); together they accounted for 61 % of the whole collection. Among them, only serotype 6A is not included in the currently used PCV10 (GSK), but it is included in PCV10 (SII, also known as PNEUMOSIL) and PCV13 (Pfizer). The most prevalent non-PCV13 serotype was serotype 46 (*n*=6, all belong to GPSC26), followed by serotype 9L (*n*=5, four belonged to GPSC627 and one to GPSC793). Due to the small sample size, we cannot confidently detect significant changes in serotype prevalence over the collection years. However, plotting the number of isolates over years by serotype with more than ten isolates, we observed declining trends in PCV10 (GSK) serotypes 14 and 6B (Fig. S1A). Serotype prevalence by clinical manifestation is summarized in Table S1.

A phased introduction of PCV10 (GSK) throughout Nigeria commenced in December 2014, but PCV uptake remained low during the study period. Overall, the projected serotype coverage for PCV10 (GSK), PCV10 (SII), PCV13 (Pfizer), PCV15 (Merck), PCV20 (Pfizer), PCV24 (Merck) and IVT-25 (Inventprise) was 67 % (*n*=129), 78 % (*n*=150), 82 % (*n*=157), 83 % (*n*=159), 85 % (*n*=163), 88 % (*n*=168) and 80 % (*n*=153), respectively. PCV15 and PCV20 are not approved for use in children, while PCV24 and IVT-25 have not been licensed at the time of writing. The serotype coverage of PCVs by clinical manifestation is summarized in Table 1. We observed high serotype coverage by PCV10 (GSK) among the disease-causing isolates. As compared to PCV10 (GSK), the PCV10 (SII, PNEUMOSIL) formulation, which includes prevalent serotypes 6A and 19A but not serotypes 4 or 18C, showed higher coverage overall and among all clinical manifestation groups, except for equal serotype coverage for the meningitis group. Serotype coverage for isolates from meningitis was the same for both PCV10 (GSK) and PCV10 (SII, PNEUMOSIL). Among the currently available PCVs, PCV13 has the highest serotype coverage and its additional coverage to PCV10 (SII, PNEUMOSIL) is due to the inclusion of serotype 18C. Our findings suggest that the currently available vaccines cover most of the circulating serotypes in Nigeria and a wider use of the vaccines is very likely to reduce child death caused by pneumococcal disease. In addition, the lower cost of PCV10 (SII, PNEUMOSIL) [29], which offers high coverage of circulating serotypes, provides great potential for including pneumococcal vaccines in routine childhood immunizations in a more sustainable fashion.

**Table 1.** PCV coverage of 192 pneumococcal isolates from Nigeria between 2014 and 2018 by clinical manifestation

Clinical manifestation		No. of isolates (%)						
	*n*	PCV10 (GSK)	PCV10 (SII)	PCV13	PCV15	PCV20	PCV24	IVT-25
Pneumonia	67	44 (66)	54 (81)	56 (84)	56 (84)	57 (85)	58 (87)	53 (79)
Bacteraemia	53	37 (70)	44 (83)	44 (83)	46 (87)	47 (89)	48 (91)	44 (83)
Meningitis	40	25 (63)	25 (63)	29 (73)	29 (73)	31 (78)	33 (83)	29 (73)
Sepsis	28	20 (71)	23 (82)	24 (86)	24 (86)	24 (86)	25 (89)	24 (86)
Other clinical manifestations	4	3 (75)	4 (100)	4 (100)	4 (100)	4 (100)	4 (100)	3 (75)
Overall	192	129 (67)	150 (78)	157 (82)	159 (83)	163 (85)	168 (88)	153 (80)

### Pneumococcal lineages

**Fig. 2.**
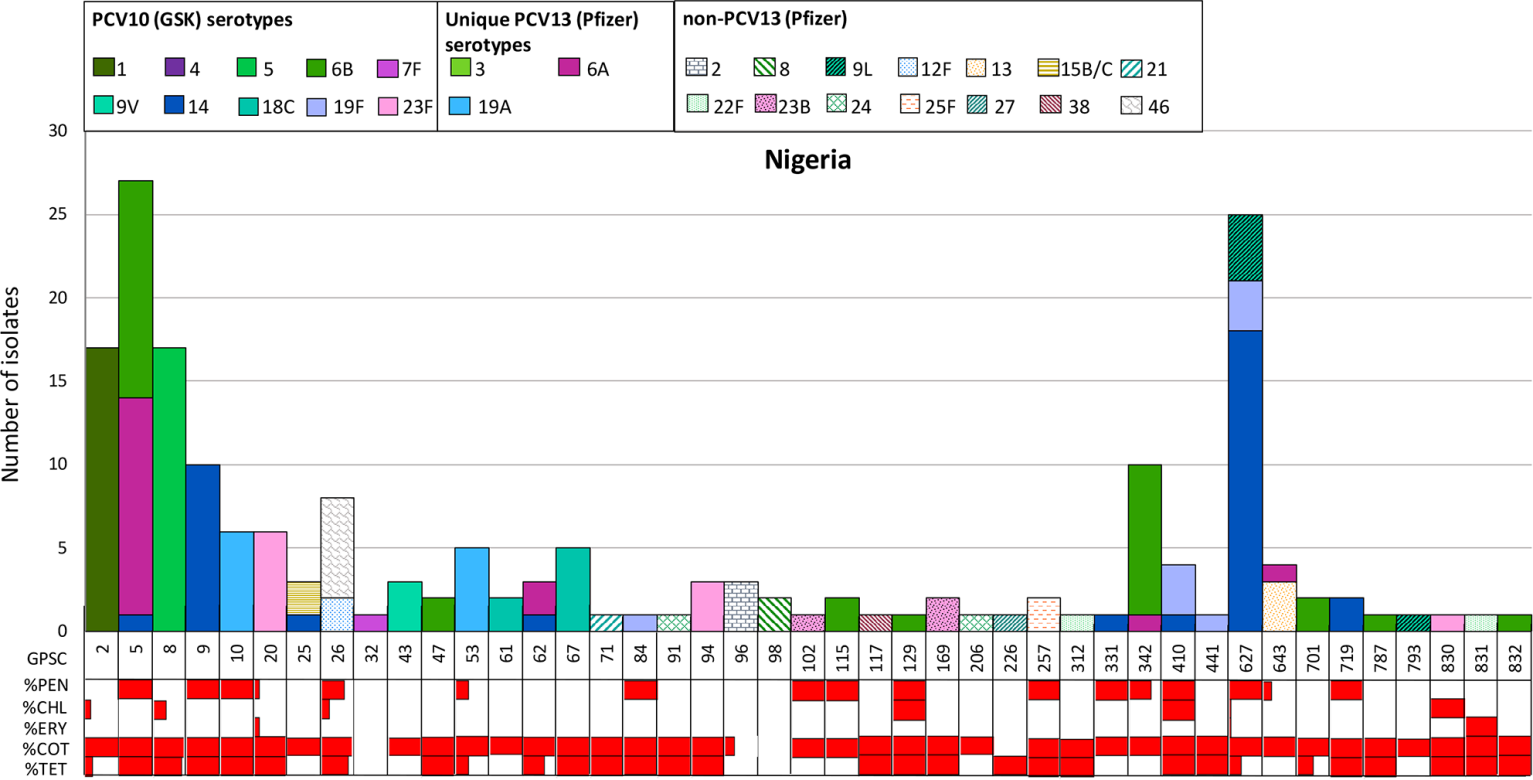
The distribution of pneumococcal lineages or Global Pneumococcal Sequence Clusters (GPSCs) by serotype composition (solid colour: PCV13 serotypes, pattern colours: non-PCV13 serotypes) and predicted antibiotic resistance profile (horizontal red bars) from genome data using a resistance detection pipeline developed by CDC [19–23 ].

Among the 192 isolates, the most predominant six pneumococcal lineages were GPSC5 (14 %, *n*=27), GPSC627 (13 %, *n*=25), GPSC2 (9 %, *n*=17), GPSC8 (9 %, *n*=17), GPSC9 (5 %, *n*=10) and GPSC342 (5 %, *n*=10), which together accounted for 55 % (106/192) of the collection (Fig. 1). Most of these isolates (83 %, 88/106) expressed serotypes that are included in PCV10 (GSK). The major non-PCV10 (GSK) serotypes in these six predominant lineages were serotype 6A expressed by GPSC5 (*n*=13) and GPSC342 (*n*=1), and serotype 9L by GPSC627 (Fig. 2). According to the GPS database (*n*=26 100, last accessed in April 2021) and pubMLST (*n*=72 521, last accessed in April 2021), GPSC627 is a locally circulating lineage in Nigeria that was uncommon elsewhere. In contrast, GPSC2, GPSC5, GPSC8 and GPSC9 were globally spreading lineages [17]. GPSC342 is a small lineage that has been documented in Bangladesh (*n*=2), Nigeria (*n*=10), Israel (*n*=3), Pakistan (*n*=1) and The Gambia (*n*=1) according to both the GPS and PubMLST databases (last accessed in January 2022). Because of the small sample size, any significant changes in prevalence of GPSCs were undeterminable.

Of the 43 GPSCs, 21 expressed purely PCV10 (GSK) serotypes, 17 only non-PCV10 (GSK) serotypes and five (GPSC5, 25, 62, 342, 627) expressed both (Fig. 2). There is evidence that following a vaccination programme, the pneumococcal population can evolve and adapt to the vaccine-selective pressure through two mechanisms: (1) an increase in lineages that only express non-vaccine serotypes (e.g. the largest non-vaccine serotype lineage GPSC26 expressing 12F and 46 in this study), and (2) expansion of non-vaccine-type component within a lineage that expresses both vaccine and non-vaccine serotypes [29 ]. As the continuous use of PCV10 (GSK) may result in serotype replacement, we described the lineages with potential to mediate serotype replacement below.

GPSC26 (CC989) expressing serotypes 12F and 46 is the largest non-PCV10 (GSK) serotype lineage found in this study, followed by GPSC10 expressing serotype 19A. GPSC26 is mainly found in Africa in both the GPS (69 %, 299/434) and PubMLST (69 %, 235/340) databases. It usually expresses serotype 12F, which has a high invasive disease potential [17, 30]. The *cps* gene clusters of serotypes 12F and 46 are almost identical with only differences in the composition of IS transpose genes [31 ]. Invasive disease potential has not been evaluated for serotype 46 due to its scarcity; it is likely that it also has a high invasive disease potential due to its genetic similarity to 12F. GPSC26 is also one of the top ten lineages found to mediate serotype replacement at an international level [32 ]. In The Gambia, an increase in serotype 12F was observed after the introduction of PCV13; such increase was mainly driven by GPSC26 [32 ]. Serotype 12F is only included in PCV20, PCV24 and IVT-25. There is no vaccine under development that will include serotype 46, but it is possible that vaccines against serotype 12F would have cross-protection against serotype 46. The use of PCV10 (GSK) or PCV13 may lead to an expansion of this lineage, which requires close monitoring in Nigeria.

Of the five GPSCs (GPSC5, 25, 62, 342, 627) with both vaccine and non-vaccine types, three (GPSC5, 62, and 342) are composed of PCV10 (GSK) vaccine types plus serotype 6A. The use of PCV10 (SII, PNEUMOSIL), PCV13 and higher-valent PCVs is likely to decrease serotype 6A within these lineages. The other two lineages, GPSC25 and GPSC627, both express vaccine serotypes plus non-vaccine serotypes 15B/C and 9L, respectively. Serotype 15B/C has a low invasive disease potential and is more frequently expressed by pneumococci colonizing healthy children, while the invasive disease potential for serotype 9L is uncertain [17, 30 ]. Serotypes 9N and 9L are highly similar in *cps* gene cluster and structure, but are more significantly different in sequence from 9V [31 ]. The high genetic similarity may suggest cross-protection against 9L capsule from PCV24 which includes 9N, but any cross-protection effect from 9V (included in all PCVs) remains unknown [33].

### Antibiotic resistance

**Fig. 3.**
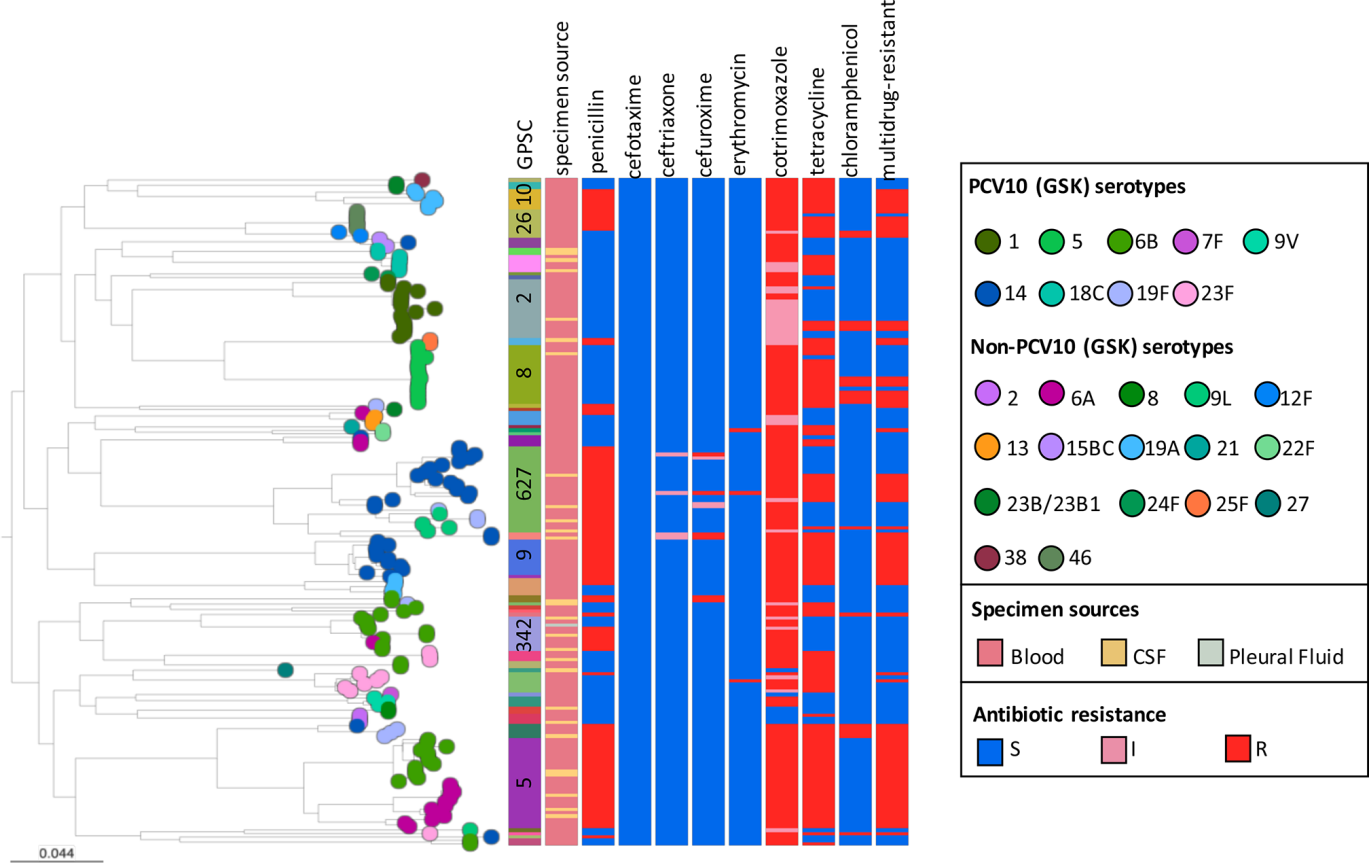
Phylogeny of 192 pneumococcal isolates from Nigeria, 2014–2016. The antimicrobial susceptibilities are predicted from genome data using a resistance detection pipeline developed by CDC [19–23 ]. Interactive figures can be visualized at https://microreact.org/project/GPS_Nigeria.

Of 192 isolates, 97 % (*n*=187) had at least one antibiotic resistance determinant and 44 % (85/192) were predicted to be multidrug-resistant (non-susceptible to at least three classes of antibiotics) (Table 2). Nearly all (185/192) isolates were cotrimoxazole non-susceptible and approximately half of the isolates were penicillin non-susceptible (PNS). However, resistance to erythromycin is low (2 %, 3/192), making macrolide antibiotics good options to treat pneumococcal disease in Nigeria. Among GPSCs with at least five isolates, four (GPSC5, 9, 10 and 627) were 100 %(68/68) PNS and four [GPSC5 (100 %, 27/27), GPSC9 (100 %, 10/10), GPSC10 (100%, 6/6) and GPSC26 (88 %, 7/8)] exhibited multidrug resistance (Fig. 3). It is of note that 36 % (9/25) of GPSC627 isolates were multidrug-resistant, with a few isolates predicted to be non-susceptible to cefuroxime (*n*=5) and ceftriaxone (*n*=2).

**Table 2.** The prevalence of antibiotic resistance of 192 pneumococcal isolates from Nigeria, 2014–2018

Antibiotic*	No. of resistant isolates	Percentage of resistance
Penicillin	99	52 %
Chloramphenicol	19	10 %
Cotrimoxazole	185	96 %
Erythromycin	3	2 %
Tetracycline	123	64 %
Multidrug-resistant	85	44 %

*The predicted MIC values were interpreted using CLSI guidelines (2014, M100-S24). For penicillin, the meningitis cut-off was used (>0.06 mg l^−1^ was categorized as resistant).

### Characterization of lineages causing disease among patients with sickle cell diseases

Of 37 pneumococcal isolates from children with sickle cell diseases or traits, 15 serotypes expressed by 19 GPSCs were detected. The most prevalent serotypes were serotypes 6B (*n*=8) and 6A (*n*=6), followed by 12 other serotypes with three or fewer isolates. Both serotypes 6A and 6B were expressed by the most prevalent lineage, GPSC5, in this subset. In addition, 6A was also found in GPSC62 (*n*=1) while 6B was found in GPSC342 (*n*=2), GPSC115 (*n*=1), and GPSC129 (*n*=1).

Compared with children without sickle cell disease, there was no significant difference in clinical manifestation or prevalence of antibiotic resistance among pneumococcal isolates collected from children with sickle cell disease (Table 3). Serotype coverage by different PCV formulations was similar between children with and without sickle cell disease, except that IVT-25 showed higher serotype coverage in children without sickle cell disease [83 % (129/155) vs 65 % (24/37), *P*=0.021]. These findings highlight that the same PCV formulation (except for IVT-25) and treatment options would be effective for both populations. Similar to the use of PCV in healthy children, PCV has proven to effectively reduce IPD among children with sickle cell disease [34 ]. Considering the high potential of occurrence of serotype replacement [35], continuous surveillance of this highly vulnerable group of children as part of the overall pathogen surveillance is needed. Penicillin is usually used as a prophylaxis for children with sickle cell disease, but the prevalence of penicillin resistance was only slightly higher in the sickle cell disease group, without statistical significance (*P*=0.584) (Table 3). Serotype 8, expressed by GPSC98, and serotype 13 by GPSC643 were more frequently found in children with sickle cell disease, but after adjusting for multiple testing, the differences were not statistically significant (Tables S3 and S4).

**Table 3.** Comparison of PCV coverage between children with (*n*=37) and without (*n*=155) traits of or confirmed sickle cell disease

	Sickle cell (*n*=37)	Non-sickle cell (*n*=155)	P*
Mean age (years) (range)	1.4 (0–5)	1.2 (0–10)	0.374
Male	21 (57 %)	106 (68 %)	0.178
**Clinical manifestation**			
Pneumonia	14 (38 %)	53 (34 %)	0.704
Bacteraemia	8 (22 %)	45 (29 %)	0.419
Meningitis	8 (22 %)	32 (21 %)	1.000
Sepsis	5 (14 %)	23 (15 %)	1.000
Others	2 (5 %)	2 (1 %)	0.168
**PCV coverage**			
PCV7	16 (43 %)	78 (50 %)	0.469
PCV10 (GSK)	20 (54 %)	109 (70 %)	0.079
PCV10 (SII)	27 (73 %)	123 (79 %)	0.385
PCV13	27 (73 %)	130 (84 %)	0.154
PCV15	27 (73 %)	132 (85 %)	0.091
PCV20	29 (78 %)	134 (86 %)	0.212
PCV24	29 (78 %)	139 (90 %)	0.092
IVT-25	24 (65 %)	129 (83 %)	0.021
**Antibiotics**			
Penicillin	21 (57 %)	78 (50 %)	0.584
Chloramphenicol	5 (14 %)	14 (9 %)	0.375
Erythromycin	1 (3 %)	2 (1 %)	0.476
Cotrimoxazole	35 (95%)	150 (97%)	0.622
Tetracycline	21 (57 %)	102 (66 %)	0.342
Multidrug resistance†	17 (46 %)	68 (44 %)	0.855

*Categorical variables were compared using Fisher’s Exact Test while continuous variables such as age were compared by Student’s t-test.

†Multidrug resistance is defined by a pneumococcal isolate predicted to be non-susceptible to ≥3 classes of antibiotics.

In summary, the high circulation of vaccine-type serotypes among disease-causing isolates strongly suggests that the currently available vaccines, especially those including prevalent serotypes 6A and 19A, could be an effective preventive measure against pneumococcal diseases among children, with or without sickle cell disease, in Nigeria. The disease-causing pneumococcal lineages in Nigeria are a mix of globally spreading and locally unique lineages. After the introduction of PCV10 (GSK), GPSC5 expressing serotype 6A, GPSC10 (19A), GPSC26 (12F and 46) and GPSC627 (9L) are high-risk lineages that could persist and potentially expand under PCV10-selective pressure. Due to the low macrolide resistance rate detected, macrolide antibiotics, such as erythromycin, could be an effective option to treat pneumococcal disease in Nigeria. However, appropriate use of macrolide antibiotics should be vigilantly monitored to prevent the potential increase in macrolide resistance.

## CAPIBD Consortium authors^†^

**Table:** 

S/No.	Name	Email
1.	Dr Zainab Adamu Haruna	zadam61@yahoo.com
2.	Dr Sadiq Abdussalam Ibrahim	sadeeqcapibd@gail.com
3.	Dr Salisu Ya’u Sulaiman	sysulaiman9@gmail.com
4.	Dr Shamsuddeen Sani	deensani@yahoo.com
5.	Dr Munir Sani	munirbinsani85@gmail.com
6.	Dr Sadiya Muhammad Sagagi	sadipotter2000@yahoo.com
7.	Dr Sani Abdullahi Yunusa	saniyunusa2014@gmail.com
8.	Dr Ubaidullah Nour Hanga	nourhangaubaidullah@gmail.com
9.	Dr Aliyu Baban Maryam	aliyubaba17@gmail.com
10.	Dr Rukayya Nasir Sani	ruqayyanasir@gmail.com
11.	Dr Jamila Bashir	bashirjamila667@gmail.com
12.	Dr Abubakar Shehu Gezawa	sagezawa@gmail.com
13.	Dr Muhammad Abdullahi Umar	mumar@yumsuk.edu.ng
14.	Dr Habiba Isah Ladu	bibals2000@yahoo.com
15.	Dr Musa Abdullahi Zango	musaabdullahiz@yahoo.com
16.	Dr Fatima Ele Aliyu	ellealiyu7322@gmail.com
17.	Dr Zakiyya Sadiq Abubakar	zakiyyasabubakar@yahoo.com
18.	Dr Abdurrauf Sani Yahaya	asaniyyahaya@gmail.com
19.	Dr Muhammad Bello	sirbello84@gmail.com
20.	Dr Bilkisu Badamasi	bilkisbadamasi@yahoo.com
21.	Dr Hajara Kabir Shehu	hajarahkshehu16@gmail.com
22.	Dr Kabiru Hamisu Haruna	kabiruharuna86@gmail.com

## Supplementary Data

Supplementary material 1
