# Supplementary material for: Molecular characterization of Streptococcus pneumoniae causing disease among children in Nigeria during the introduction of PCV10 (GSK)
Source: Microb Genom. 2023 Sep 15;9(9):001094. doi: 10.1099/mgen.0.001094 (PMC10569732; doi:10.1099/mgen.0.001094)
Supplement: Supplementary material 1 [file mgen-9-1094-s001.pdf]

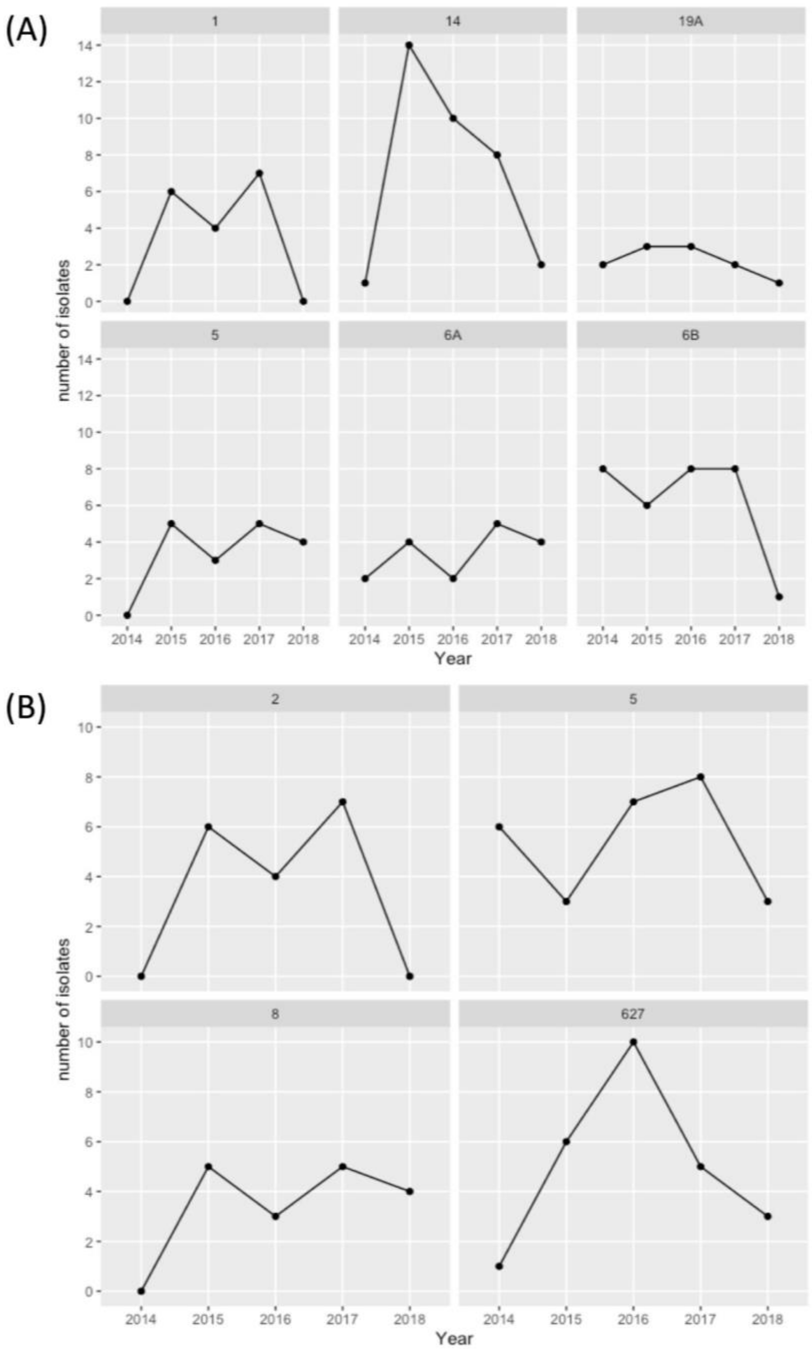

Figure S1. Number of pneumococcal isolates belong to prevalent serotypes (A) and GPSCs (B) with >10 isolates from Nigeria, 2014-2018. Serotypes 1, 5, 6B, and 14 are included in PCV10 (GSK) which was introduced in December 2014 while serotypes 6A and 19A are not included in PCV10 (GSK). GPSC2 only expresses PCV10 serotypes 1; GPSC5 expresses both PCV10 serotypes 6B and 14 and non-PCV10 serotype 6A; GPSC8 expresses serotype 5 only; GPSC627 expressed both PCV10 (GSK) serotypes 14 plus 19F and non-PCV10 (GSK) serotype 9L.

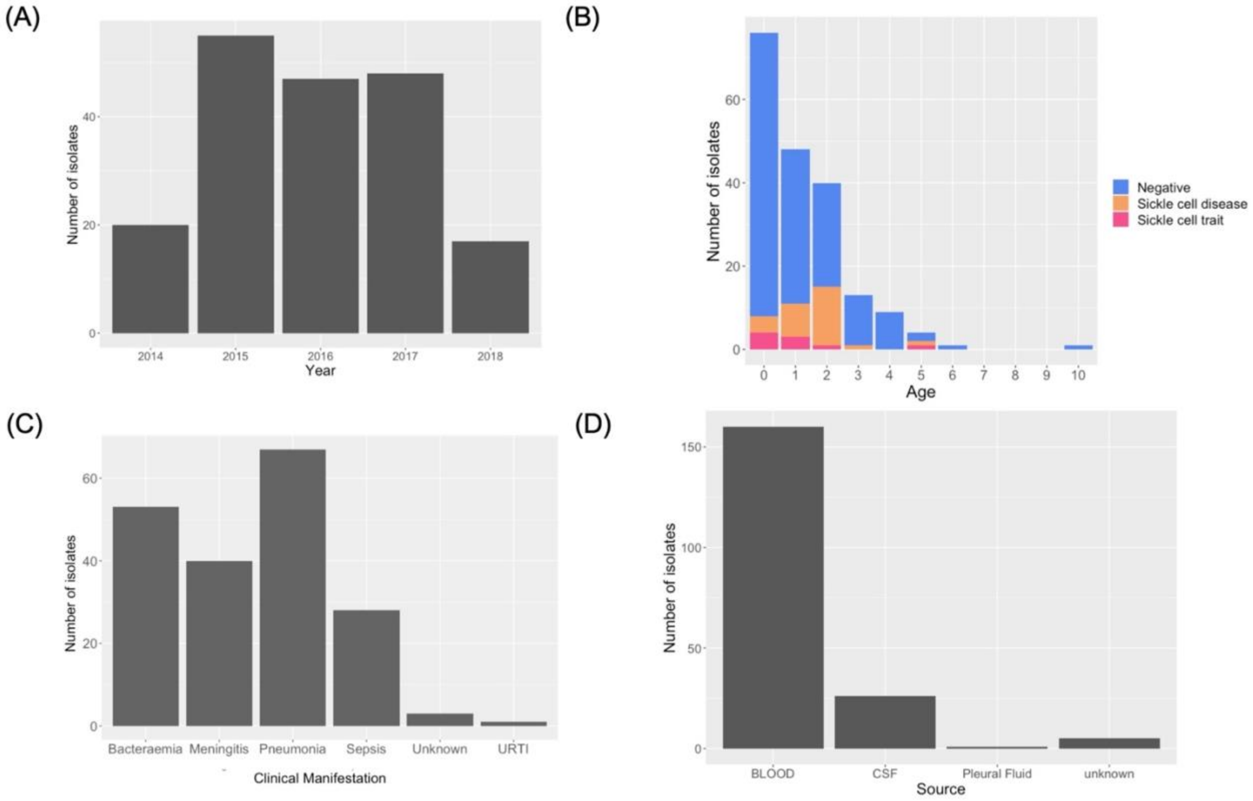

Figure S2 The pneumococcal collection from Kano (n=189) and Abuja (n=3) states Nigeria between 2014 and 2018 by A) year, B) age, C) clinical manifestation and D) source of clinical samples.

Table S1. Serotype prevalence of 192 pneumococcal isolates from NIgeria collected between 2014 and 2016 by clinical manifestation

Serotypes	Pneumonia	Bacteraemia	Meningitis	Sepsis	Other clinical manifestations	Overall
**PCV10[1]**						
14	10	13	6	6	-	35
6B	11	8	7	5	-	31
1	9	4	1	2	1	17
5	5	7	2	2	1	17
23F	2	3	-	4	1	10
18C	2	-	4	1	-	7
19F	3	-	5	-	-	8
9V	1	2	-	-	-	3
7F	1	-	-	-	-	1
**PCV13 additionals[2]**						
6A	5	4	4	3	1	17
19A	7	3	-	1	-	11
**non-PCV13**						
46	4	1	1	-	-	6

9L	1	3	1	-	-	5
2	-	1	2	-	-	3
13	2	1	-	-	-	3
23B	-	-	2	1	-	3
8	1	-	1	-	-	2
12F	-	1	1	-	-	2
15B/C	1	-	-	1	-	2
22F	-	2	-	-	-	2
24F	-	-	-	2	-	2
25F	-	-	2	-	-	2
21	1	-	-	-	-	1
27	-	-	1	-	-	1
38	1	-	-	-	-	1

[1]No serotype 4 was observed in this collection.

[2]No serotype 3 was observed in this collection.

Table S2. Predicted β-lactam MIC values of pneumococcal lineages with more than five isolates from Nigeria, 2014-2018

GPSCs	No. of isolates	MIC median (range) in μg/ml					
		Penicillin	Amoxicillin	Meropenem	Cefotaxime	Ceftriaxone	Cefuroxime
GPSC5[a]	27	0.12 (0.12/0.25)	0.06 (0.03-0.06)	≤0.06 (≤0.06)	0.12 (0.12)	≤0.5 (≤0.5)	≤0.5 (≤0.5)
GPSC627[a]	25	0.5 (0.25-1)	0.25 (0.06-0.5)	0.12 (0.06-0.25_	0.12 (0.12-0.5)	0.5 (0.5-1)	0.5 (0.5-2)
GPSC2	17	0.03 (0.03-0.06)	≤0.03 (≤0.03)	≤0.06 (≤0.06)	0.06 (0.06-0.12)	≤0.5 (≤0.5)	≤0.5 (≤0.5)
GPSC8	17	≤0.03 (≤0.03)	≤0.03 (≤0.03)	≤0.06 (≤0.06)	≤0.06 (≤0.06)	≤0.5 (≤0.5)	≤0.5 (≤0.5)
GPSC9[a]	10	0.25 (0.12-0.25)	0.12 (0.03-0.25)	≤0.06 (≤0.06)	0.12 (0.12-0.25)	≤0.5 (≤0.5)	≤0.5 (≤0.5)
GPSC342	10	0.12 (0.03-0.12)	0.03 (0.03-0.12)	0.06 (0.06-0.12)	0.12 (0.06-0.12)	≤0.5 (≤0.5)	≤0.5 (≤0.5)
GPSC26	8	0.12 (0.03-0.12)	0.12 (0.03-0.12)	≤0.06 (≤0.06)	≤0.06 (≤0.06)	≤0.5 (≤0.5)	≤0.5 (≤0.5)
GPSC10[a]	6	0.5 (0.5)	0.25 (0.25)	0.12 (0.12)	0.12 (0.12)	≤0.5 (≤0.5)	≤0.5 (≤0.5)
GPSC20	6	0.03 (0.03-0.25)	0.03 (0.03-0.25)	0.06 (0.06-0.12)	0.06 (0.06-0.12)	≤0.5 (≤0.5)	≤0.5 (≤0.5)

Resistance to penicillin was defined as MIC of ≥0.12 µg/ml, amoxicillin as MIC of ≥8 µg/ml, meropenem as MIC of ≥1 µg/ml, cefotaxime, ceftriaxone, cefuroxime as MIC of ≥2 µg/ml according to Clinical and Laboratory Standards Institute guideline.

[a]Pencillin-resistant lineages that had 100% isolates resistant to penicillin using CLSI meningitis breakpoint (≥0.12 μg/ml)

Table S3 Comparison of serotype prevalence between children with sickle cell disease (n=37) and without (n=155).

Serotype	Sickle cell (n=37)	Non-sickle cell (n=155)	p	Adjusted p[a]
13	3 (8%[b])	0	0.007*	0.175
8	2 (5%)	0	0.036*	0.450
14	3 (8%)	32 (21%)	0.097	0.650
6A	6 (16%)	11 (7%)	0.104	0.650
38	1 (3%)	0	0.193	0.846
5	1 (3%)	16 (10%)	0.203	0.846
6B	8 (22%)	23 (15%)	0.325	0.875
46	2 (5%)	4 (3%)	0.327	0.875
24F	1 (3%)	1 (0.6%)	0.349	0.875
18C	0	7 (5%)	0.350	0.875
23F	3 (8%)	7 (5%)	0.409	0.930
9V	1 (3%)	2 (1%)	0.476	0.992
19A	1 (3%)	10 (6%)	0.694	1
22F	0	2 (1%)	1	1
9L	1 (3%)	4 (3%)	1	1

1	3 (8%)	14 (9%)	1	1
19F	1 (3%)	7 (5%)	1	1
2	0	3 (2%)	1	1
27	0	1 (0.6%)	1	1
23B	0	3 (2%)	1	1
12F	0	2 (1%)	1	1
15B/15C	0	2 (1%)	1	1
25F	0	2 (1%)	1	1
21	0	1 (0.6%)	1	1
7F	0	1 (0.6%)	1	1

[a]Multiple testing was adjusted by using the Benjamin-Hochberg false discovery rate of 5%.

[b]Percentages may not total 100 due to rounding

Table S4 Comparison of GPSC prevalence between children with sickle cell disease (n=37) and without (n=155).

GPSC	Sickle cell (n=37)	Non-sickle cell (n=155)	p	Adjusted p[a]
643	3 (8%[b])	1 (0.6%)	0.023	0.781
98	2 (5%)	0	0.036	0.781
342	4 (11%)	6 (4%)	0.103	1
117	1 (3%)	0	0.193	1
206	1 (3%)	0	0.193	1
129	1 (3%)	0	0.193	1
8	1 (3%)	16 (10%)	0.203	1
20	2 (5%)	4 (3%)	0.326	1
115	1 (3%)	1 (0.6%)	0.349	1
627	3 (8%)	22 (14%)	0.422	1
5	7 (19%)	20 (13%)	0.428	1
43	1 (3%)	2 (1%)	0.476	1
62	1 (3%)	2 (1%)	0.476	1
94	1 (3%)	2 (1%)	0.476	1
410	1 (3%)	3 (2%)	0.578	1

67	0	5 (3%)	0.585	1
53	0	5 (3%)	0.585	1
26	2 (5%)	6 (4%)	0.652	1
9	1 (3%)	9 (6%)	0.690	1
2	3 (8%)	14 (9%)	1	1
10	1 (3%)	5 (3%)	1	1
831	0	1 (0.6%)	1	1
312	0	1 (0.6%)	1	1
61	0	2 (1%)	1	1
96	0	3 (2%)	1	1
832	0	1 (0.6%)	1	1
226	0	1 (0.6%)	1	1
102	0	1 (0.6%)	1	1
701	0	2 (1%)	1	1
719	0	2 (1%)	1	1
25	0	3 (2%)	1	1

830	0	1 (0.6%)	1	1
331	0	1 (0.6%)	1	1
257	0	2 (1%)	1	1
787	0	1 (0.6%)	1	1
84	0	1 (0.6%)	1	1
441	0	1 (0.6%)	1	1
71	0	1 (0.6%)	1	1
793	0	1 (0.6%)	1	1
169	0	2 (1%)	1	1
32	0	1 (0.6%)	1	1
47	0	2 (1%)	1	1
91	0	1 (0.6%)	1	1

[a]Multiple testing was adjusted by using the Benjamin-Hochberg false discovery rate of 5%.

[b]Percentages may not total 100 due to rounding.
